# Expectation of antibiotics amongst owners of dogs and/or cats during non-routine visits to veterinary clinics in Singapore: a cross-sectional study

**DOI:** 10.3389/fvets.2024.1491054

**Published:** 2024-11-18

**Authors:** Seema Aithal, Huiling Guo, Boon Han Teo, Timothy Chua, Zoe Jane-Lara Hildon, Angela Chow

**Affiliations:** ^1^Tan Tock Seng Hospital, Singapore, Singapore; ^2^Singapore Veterinary Association, Singapore, Singapore; ^3^Beecroft Animal Specialist and Emergency Hospital, Singapore, Singapore; ^4^Saw Swee Hock School of Public Health, National University of Singapore, Singapore, Singapore; ^5^Lee Kong Chian School of Medicine, Nanyang Technological University, Singapore, Singapore

**Keywords:** antimicrobial resistance, antibiotic expectation, One Health, pet owners, survey

## Abstract

**Objectives:**

The present study aimed to assess the factors associated with pet owners’ expectations for receiving antibiotics for their pet dogs and/or cats and the factors associated with pets (dogs and/or cats) receiving antibiotics during non-routine veterinary clinic consultations in Singapore.

**Methods:**

A cross-sectional study was conducted on consenting pet owners who attended 16 veterinary clinics in Singapore, between March and December 2023. An online survey measured participants’ knowledge of antibiotic use, prior antibiotic use experience, expectation for antibiotics and receipt of antibiotics during the last non-routine clinic consultation for their pets. Multivariable logistic regression models were used to determine the factors associated with expectation of antibiotics and receipt of antibiotics.

**Results:**

Among the 821 pet owners, over one-in-four (27.5%) expected antibiotics. Owners with prior antibiotic use (adjusted OR 5.18, 95%CI 2.85–9.42) and poor knowledge of antibiotic use (adjusted OR 1.69, 95% CI 1.21–2.35) were 5 times and nearly 2 times as likely as owners without prior antibiotic use and those with good knowledge of antibiotic use respectively, to expect antibiotics for their pets. After adjusting for potential confounders, owners who expected antibiotics (adjusted OR 12.09, 95% CI 7.83–18.68) and had prior antibiotic use for their pets (adjusted OR 8.57, 95% CI 4.75–15.47) were more likely to receive antibiotics for their pets.

**Conclusion:**

Factors which significantly influenced expectation of antibiotics in pet owners included poor knowledge of antibiotic use and prior usage of antibiotics. This highlights the importance of effective communication by veterinarians to mitigate pet owners’ expectations to address inappropriate antibiotic prescribing.

## 1 Introduction

Antimicrobial resistance (AMR) is a longstanding problem which poses a serious threat to human health (1). A One Health approach promoting collaboration between the human, animal and environment sectors has been strongly espoused by the World Health Organization (WHO) to address this wicked problem (2).

It is well known that overuse or misuse of antibiotics can contribute to AMR in humans (3). According to human studies, patients’ expectations for antibiotics are major influencers of inappropriate antibiotic prescribing (4, 5). Similarly, veterinarians are more likely to prescribe antibiotics when pet owners are perceived to be expecting them for their pets (6–9). Owners’ expectation for some kind of medication, preferably antibiotics to treat their pet dogs and cats coupled with poor understanding of the risks associated with antibiotic use have been reported as drivers of inappropriate antibiotic prescribing (6–8). But these expectations are seldom made explicit during veterinary consultations and only a minority of pet owners directly pushed for antibiotics because they were risk averse and did not want their pets to suffer (9).

Growing pet ownership numbers combined with the close physical proximity between owners and their pets due to deep attachment favors AMR transmission in both directions (10) emphasizing the urgent need for the prudent use of antibiotics in pets. While insights about pet owners’ opinions and expectations surrounding the use of antibiotics have mostly been derived from studies conducted in Western countries (6–9, 11, 12), there is very limited data on this topic from a Southeast Asian context where pet populations have been steadily increasing (13).

While it is foreseeable that pet owners’ expectation of antibiotics might influence antibiotic prescribing for their ‘fur babies’ (7), little is known about the determinants of their expectation for antibiotics. Hence, we aimed to investigate (1) factors associated with pet owners’ expectations for receiving antibiotics for their pet dogs and/or cats and (2) factors associated with pets (dogs and/or cats) receiving antibiotics during non-routine veterinary clinic consultations in Singapore. Insights gathered from a local context may be helpful in developing strategies to curb inappropriate use of antibiotics in pets.

## 2 Methods

### 2.1 Study design and sampling frame

We conducted a cross-sectional study on consenting pet owners who attended 16 veterinary clinics in Singapore, between March 2023 and December 2023. The sampling frame included around 100 veterinary clinics licensed under the Animal and Veterinary Service (AVS), Singapore at the point of data collection. The clinics were first stratified by location (North, South, East, West and Central) and then according to the size of the practice (solo/small group–2 or less clinics under same name and large chain–more than 2 clinics) to account for diverse organizational practices relating to pet management.

To date, no study quantifying the effect of factors influencing pet owners’ expectations of antibiotics for their pet dogs and cats has been reported. From a previous local study on human health assessing factors associated with antibiotic expectation by patients with uncomplicated upper respiratory tract infection, the significant factors had odds ratios of 1.28 and above (14). Assuming a two-sided test with a power of 80%, an alpha level of 5% and a 1:1 ratio of expectation vs. non-expectation of antibiotics, a minimum sample size of 680 will be adequate to detect a minimum odds ratio of 1.28 in factors (with a prevalence of ≥25% in the group who do not expect antibiotics) influencing owners’ expectation for antibiotics in companion animals. This study is part of a larger study whose target sample was 1,074 and assuming 62% of all veterinary visits to be non-routine consultations based on observations from a prior study investigating preventive-medicine consultations in small animal practices in the UK (15), we will be able to achieve the minimum sample size required to detect effects with odds ratios of 1.28 and above in factors influencing owners’ expectations for antibiotics in companion animals.

To reach the target sample size, an equal number of eligible pet owners (aged 21 years and above) of dogs and/or cats were recruited consecutively from 3 to 4 clinics purposively sampled from each of the five zones to ensure a good representation of solo and large veterinary general practices from all regions of Singapore. The participants were invited to complete a self-administered survey hosted on an online platform, as they brought their pets for consultation at the clinics. For the present study, we only included those visits where consultation was sought for reasons other than routine check-ups, vaccination, obesity, osteoarthritis/mobility-related and metabolic issues, classified as non-routine visits.

The study was approved by the Domain Specific Review Board, National Healthcare Group, Singapore (Reference Number: 2021/00769). Consent was implied if pet owners voluntarily completed the online survey after reading the study information sheet.

### 2.2 Survey instrument

The anonymous questionnaire contained question items from surveys conducted in human health, adapted to the veterinary setting (16, 17). It comprised questions on socio-demographics (age in years, gender [male/female], ethnicity [Chinese/non-Chinese], marital status [yes/no], education [post-secondary & below as lower educated/ diploma & higher as higher educated]), type of pet(s) owned (dog, cat or both), experience as a pet dog/cat owner (<10 years/≥10 years), prior or ever use of antibiotics for their pet dog/cat, 3 items on the knowledge of the appropriate use of antibiotics for their pets (True/False/Do not know) and 9 items on the understanding of the issue of AMR in their pets (True/False/Do not know) from the WHO’s Antibiotic Resistance Multi-country Public Awareness Survey (18) adapted accordingly. Pet owners were deemed to have poor knowledge of antibiotic use, if they incorrectly answered any of the 3 items on antibiotic use for their pets. The items were “It is (not) okay to use antibiotics for my pet dog/cat that were given to another pet dog/cat, as long as they were being used to treat the same illness.” “It is (not) okay to buy the same antibiotics or request them from a veterinary practitioner (vet), if they helped my pet dog/cat get better previously when it had the same symptoms” and “Once your pet dog/cat has begun antibiotic treatment, you should only stop giving antibiotics when it has taken all of them as directed.” For AMR knowledge in pets, the total composite score was 9 based on correct responses to all 9 items and pet owners who scored less than 75th percentile (i.e., below 5) were deemed to have poor knowledge of AMR.

The survey also included questions about the reason for their pet dog/cat’s last consultation at the veterinary clinic, whether they expected antibiotics to be prescribed (e.g., At that last consultation, were you expecting antibiotics for your pet cat/dog from the veterinary practitioner (vet)?-Yes/No/Cannot remember) and if their pet dog(s)/cat(s) received antibiotics on that occasion (e.g., Did your pet cat/dog receive antibiotics from the veterinary practitioner (vet) during that consultation?-Yes/No/Cannot remember). In the present study, we defined “receipt of antibiotics” as (1) antibiotics prescribed (and sold) by the vet clinic during the consultation to be used later and/or (2) antibiotics administered onsite during the consultation. Satisfaction with the veterinarian’s decision to either administer or not administer antibiotics to their pet(s) on that occasion was assessed using a 5-point Likert scale, ranging from extremely dissatisfied to strongly satisfied (e.g., How did you feel about the decision to administer or not administer antibiotics for your pet cat/dog at that time?) (Questionnaire: Supplementary materials, Document S1).

### 2.3 Data analysis

The outcomes of interest were whether pet owners (1) expected antibiotics and (2) received antibiotics for their pets during their last non-routine veterinary consultation. We used appropriate descriptive statistics to summarize pet owners’ demographic characteristics, pet-related factors, knowledge of antibiotic use and AMR for their pets, prior/ever use of antibiotics. First, we conducted univariable analysis to inform variable selection for subsequent multivariable analysis. We included sociodemographic factors and other variables identified *a priori* as being associated with expectation for antibiotics in human studies and not specifically by pet owners from literature review. Next, we assessed the independent factors associated with pet owner’s expectation for antibiotics and receipt of antibiotics by adding variables to an initial model using multivariable logistic regression models. Collinearity among covariates was measured by means of variance inflation factor. Strongly correlated variables were excluded from the multivariable models. The Akaike’s Information Criteria (AIC), Bayesian Information Criteria (BIC) and log likelihood ratio statistic guided the selection of final model (Supplementary Tables S2A, S2B). Statistical analyses were performed using STATA 14.0 (StataCorp LLC, College Station, TX, USA).

## 3 Results

Of the 1933 eligible pet owners (adults aged 21 years and above) of dogs and/or cats who were approached, 1,080 (55.9%) completed responses were collected from 16 veterinary clinics across Singapore. For this study, we included only 821 pet owner responses based on the reason for their pet dog/cat’s last consultation with the veterinarian classified as ‘non-routine visits’.

### 3.1 Baseline characteristics

Among the 821 pet owners who sought consultation for non-routine visits, majority were females (63.7%), married (56%) and higher educated (86.2%) with a mean age of 41.1 years (SD: 12.5). Nearly two-in-three (68.3%) pet owners owned dogs (inclusive of those who owned both dogs and cats-7.6%) and 408 (49.7%) had more than 10 years of pet ownership experience. Nearly 4-in-5 pet owners (82%) reported prior use of antibiotics and almost half (47%) had poor knowledge of antibiotic use. Two-in-three pet owners (69.4%) had poor knowledge of AMR (Table 1).

**Table 1:** Logistic regression analysis of the factors associated with expectation of antibiotics amongst pet owners of dogs and/or cats who attended veterinary clinics for non-routine visit (*N* = 821).

Variables	Overall (*N* = 821)	Expected antibiotics (*N* = 226)	Did not expect antibiotics (*N* = 595)	Univariable model		Multivariable model	
				Crude OR (95% CI)	*p* value^*^	Adjusted OR (95% CI)	*p* value^*^
Age in years, mean (SD)^a^	41.1 (12.5)	39.3 (12.9)	41.7 (12.2)	0.98 (0.97–0.99)	**0.013**	–	
Gender, N(%)							
Male	298 (36.3)	74 (32.7)	224 (37.7)	Reference		Reference	
Female	523 (63.7)	152 (67.3)	371 (62.4)	1.24 (0.9–1.71)	0.192	1.21 (0.85–1.72)	0.286
Ethnic group, N(%)							
Chinese	597 (72.7)	150 (66.4)	447 (75.1)	Reference		Reference	
Non-Chinese	224 (27.3)	76 (33.6)	148 (24.9)	1.53 (1.10–2.13)	**0.012**	1.66 (1.15–2.40)	**0.007**
Education, N(%)							
Lower educated (Post-secondary & below)	113 (13.8)	41 (18.1)	72 (12.1)	1.61 (1.06–2.45)	**0.026**	1.71 (1.10–2.67)	**0.017**
Higher educated (Diploma & above)	708 (86.2)	185 (81.9)	523 (87.9)	Reference		Reference	
Marital status, N(%)							
Yes	463 (56.4)	110 (48.7)	353 (59.3)	Reference		Reference	
No	358 (43.6)	116 (51.3)	242 (40.7)	1.54 (1.13–2.09)	**0.006**	1.61 (1.16–2.22)	**0.004**
Own dogs, N(%)							
Yes	561 (68.3)	158 (69.9)	403 (67.7)	1.11 (0.79–1.54)	0.827	1.25 (0.86–1.82)	0.241
No	260 (31.7)	68 (30.1)	192 (32.3)	Reference		Reference	
Ownership duration, N(%)							
<10 years	413 (50.3)	117 (51.8)	296 (49.8)	Reference		Reference	
≥10 years	408 (49.7)	109 (48.2)	299 (50.3)	0.92 (0.68–1.25)	0.605	0.83 (0.60–1.15)	0.259
Knowledge of antibiotic use for pets^b^, N(%)							
Not Poor	435 (53.0)	101 (44.7)	334 (56.1)	Reference		Reference	
Poor	386 (47.0)	125 (55.3)	261 (43.9)	1.58 (1.16–2.16)	**0.003**	1.69 (1.21–2.35)	**0.002**
Knowledge of AMR for pets, N (%)							
Not Poor	251 (30.6)	78 (34.5)	173 (29.1)	Reference		-	
Poor	570 (69.4)	148 (65.5)	422 (70.1)	0.78 (0.56–1.08)	0.131		
Prior antibiotic use, N(%)							
Yes	675 (82.2)	212 (93.8)	463 (77.8)	4.32 (2.43–7.67)	**<0.001**	5.18 (2.85–9.42)	**<0.001**
No	146 (17.8)	14 (6.2)	132 (22.2)	Reference		Reference	
Received antibiotics, N(%)	383 (46.7)	193 (85.4)	190 (31.9)	–		–	

CI, Confidence Interval; OR, Odds Ratio. ^*^ Bolded values indicate statistical significance of *p* < 0.05.

a Age excluded in multivariable model due to high Variance Inflation Factor.

b Definition of Knowledge of antibiotic use (only stop antibiotics when completed entire course of antibiotics as directed, not to use antibiotics given to another pet and not to buy/request for previously-used antibiotics).

### 3.2 Antibiotic expectation

Overall, nearly one-in-four pet owners (27.5%) expected antibiotics during their pet dog/cat’s last non-routine veterinary clinic consultation. A higher proportion of pet owners who expected antibiotics were younger (*p* = 0.007), non-Chinese (33.6% vs. 24.9%, *p* = 0.012), not married (51.3% vs. 40.7%, *p* = 0.006) and lower educated (18.1% vs. 12.1%, *p* = 0.025). Compared to those who did not expect antibiotics, a higher proportion of pet owners who expected antibiotics received them (85% vs. 32%, *p* < 0.001). Pet ownership duration and type of pet did not differ between those who expected and did not expect antibiotics (Table 1).

### 3.3 Receipt of antibiotics

Almost half of all pet owners (46.7%) reported receiving antibiotics for their pet dog/cat’s last non-routine veterinary clinic consultation. Compared to those who did not receive antibiotics, a higher proportion of pet owners who received antibiotics had used antibiotics previously (95.6% vs. 70.6%, *p* < 0.001). A higher proportion of owners who received antibiotics during their last veterinary consultation had expected antibiotics to be prescribed (50.4%vs7.5%, *p* < 0.001). Regarding client satisfaction, a higher proportion of pet owners who received antibiotics than those who did not, reported being satisfied with the overall consult (48.1% vs. 24.9%, *p* < 0.001) (Table 2).

**Table 2:** Logistic regression analysis of the factors associated with receipt of antibiotics amongst pet owners of dogs and/or cats during non-routine veterinary clinic consultations (*N* = 821).

Variables	Overall (*N* = 821)	Received antibiotics (*N* = 383)	Did not receive antibiotics (*N* = 438)	Univariable model		Multivariable model	
				Crude OR (95% CI)	*p* value^*^	Adjusted OR (95% CI)	*p* value^*^
Age in years, mean (SD)^a^	41.1 (12.5)	40.7 (12.6)	41.4 (12.4)	0.99 (0.98–1.01)	0.395	–	
Gender, N(%)							
Male	298 (36.3)	136 (35.5)	162 (37.0)	Reference		Reference	
Female	523 (63.7)	247 (64.5)	276 (63.0)	1.07 (0.80–1.42)	0.661	0.83 (0.59–1.17)	0.291
Ethnic group, N(%)							
Chinese	597 (72.7)	275 (71.8)	322 (73.5)	Reference		Reference	
Non-Chinese	224 (27.3)	108 (28.2)	116 (26.5)	1.09 (0.80–1.48)	0.582	0.81 (0.55–1.21)	0.295
Education, N(%)							
Lower educated (Post-secondary & below)	113 (13.8)	50 (13.1)	63 (14.4)	0.89 (0.60–1.33)	0.582	0.73 (0.44–1.72)	0.227
Higher educated (Diploma & above)	708 (86.2)	333 (87.0)	375 (85.6)	Reference		Reference	
Own dogs, N(%)							
Yes	561 (68.3)	259 (67.6)	302 (69.0)	0.94 (0.71–1.26)	0.680	0.68 (0.47–0.99)	**0.043**
No	260 (31.7)	124 (32.4)	136 (31.1)	Reference		Reference	
Ownership duration, N(%)							
<10 years	413 (50.3)	192 (50.1)	221 (50.5)	Reference		Reference	
≥10 years	408 (49.7)	191 (49.9)	217 (49.5)	1.01 (0.77–1.33)	0.926	0.83 (0.59–1.17)	0.288
Prior antibiotic use, N(%)							
Yes	675 (82.2)	366 (95.6)	309 (70.6)	8.99 (5.50–15.24)	**<0.001**	8.57 (4.75–15.47)	**<0.001**
No	146 (17.8)	17 (4.4)	129 (29.5)	Reference		Reference	
Expectation of antibiotics, N(%)							
Yes	226 (27.5)	193 (50.4)	33 (7.5)	12.47 (8.30–18.74)	**<0.001**	12.09 (7.83–18.68)	**<0.001**
No	595 (72.5)	190 (49.6)	405 (92.5)	Reference		Reference	
Satisfied with visit, N(%)	293 (35.7)	184 (48.1)	109 (24.9)	–		–	

CI, Confidence Interval; OR, Odds Ratio. ^*^ Bolded values indicate statistical significance of *p* < 0.05.

a Age excluded in multivariable model due to high Variance Inflation Factor.

### 3.4 Determinants of expectation for antibiotics

Pet owners with prior antibiotic use and poor knowledge of antibiotic use were more likely to expect antibiotics for their pets. Compared with pet owners without prior antibiotic use, those with a history of prior antibiotic use (adjusted OR 5.18, 95% CI: 2.85–9.42) were significantly more likely to expect antibiotics for their pets. Pet owners with poor knowledge of antibiotic use (adjusted OR 1.69, 95% CI: 1.21–2.35) were nearly twice as likely to expect antibiotics compared to those with good knowledge of antibiotics. Furthermore, pet owners who were non-Chinese (adjusted OR 1.66, 95% CI: 1.15–2.40), lower educated (adjusted OR 1.71, 95% CI: 1.10–2.67) and not married (adjusted OR 1.61, 95% CI: 1.16–2.22) were more likely to expect antibiotics for their pets during non-routine veterinary clinic consultation (Table 1).

### 3.5 Determinants of receipt of antibiotics

After adjusting for potential confounders, owners who expected antibiotics (adjusted OR 12.09, 95% CI 7.83–18.68) and had prior antibiotic use for their pets (adjusted OR 8.57, 95% CI 4.75–15.47) were more likely to receive antibiotics for their pets compared to those pet owners who did not expect antibiotics and had no prior antibiotic use, respectively (Table 2).

## 4 Discussion

Approximately a quarter of pet owners of dogs and/or cats (27.5%) expected antibiotics to be prescribed during non-routine veterinary clinic consultations in Singapore. Our finding is lower compared to studies from Australia, US and UK (6, 11, 12) where 49–54% of pet owners expected antibiotics to be prescribed at the time of a sick visit. We also observed that Singaporean pet owners who were younger, non-Chinese, not married and having lower educational levels were more likely to expect antibiotics. Past studies have also noted that educational background and knowledge of antibiotics may influence pet owner preferences and expectations regarding antibiotic use (6, 7, 19).

We found that pet owners’ poor knowledge of antibiotic use and prior history of antibiotic use for their pets were associated with increased expectation of antibiotics after adjustment for socio-demographic (gender, ethnicity, education, marital status) and pet factors (pet type, ownership duration). Determinants of antibiotic expectation have not been reported previously in pet owners, but our results were consistent with a local study conducted among adult human patients with upper respiratory tract infection attending emergency departments during the COVID-19 pandemic (4).

Of concern, nearly half of all pet owners (47%) had poor knowledge of antibiotic use for pets and two-in-three pet owners (69.4%) demonstrated poor knowledge of AMR in our cohort. Prior studies have revealed that pet owners generally had a poor understanding of antibiotics’ ineffectiveness against viruses, possible side effects of indiscriminate use of antibiotics and the potential interspecies transmission of AMR genes between pets and humans (8, 12, 20). Tackling these knowledge gaps is vital from a One Health perspective and present as opportunities for action. Enhancing pet owners’ awareness using educational posters and pamphlets or decision aids, followed by in-depth discussions about appropriate antibiotic use and AMR, may be considered by veterinarians in their clinic settings.

We found that a higher proportion of pet owners who expected antibiotics reported that it was okay to buy the same antibiotics or request them from a veterinary practitioner, if they helped their pet cat/dog get better previously when it had the same symptoms (47.4% vs. 35.5%, *p* = 0.002). This finding highlights the need to educate pet owners on appropriate antibiotic use and AMR. Veterinarians should tailor their messages based on pet owners’ level of understanding and seek to clarify misunderstandings surrounding the use of antibiotics in simple layman’s terms. It has been previously noted that pet owners generally trust and accept their veterinarians’ expertise and were amenable to their advice if actively engaged in the decision-making process regarding their pet’s health (8, 20). Veterinarians can leverage this to inform pet owners about appropriate antibiotic use during routine clinic consultations which may enable greater compliance from pet owners. Studies have reported the importance of effective communication and engagement of pet owners as key stakeholders for facilitating appropriate antimicrobial use in pets (6, 11, 21).

The present study also revealed that pet owners who expected antibiotics were more likely to receive antibiotics for their pets which is similar to the results from human studies on patients attending emergency departments and primary care clinics (4, 5). A qualitative study from UK (8) reported that veterinarians faced tacit pressures from pet owners for a tangible outcome, usually in the form of prescribed antibiotics or they succumbed to economic pressures due to client attrition. On the other hand, it has been suggested that pet owners’ decision to seek antibiotics for their pets is due to the unconditional love and responsibility along with the perceived vulnerability of their pets (7). This precautionary approach may explain their unwarranted expectation for antibiotics which acts as a modifiable driver for AMR.

Our study had some limitations. The study findings are purely associational due to the cross-sectional nature of our study design and does not imply causation (22). Data on perceived severity of the illness and chronic conditions which may necessitate the use of antibiotics were not collected and hence we cannot distinguish whether pet owners’ expectation of antibiotics for their pet was appropriate or not. Social desirability bias cannot be ruled out as pet owners were recruited by study team members during clinic visits, but this was likely to be minimal as the survey was anonymous, online and self-administered. Our study sample may not be representative as we purposively sampled veterinary clinics by practice types and locations in Singapore, and recruited pet owners in-person to ensure that they are indeed owners of dogs and cats. Nonetheless, the pet ownership profile in our survey is similar to those reported by studies conducted in Western countries (6, 11, 20). Despite these limitations, to our knowledge, this is the first study to explore the factors associated with pet owners’ expectation for antibiotics, which is timely considering the increasing emphasis on AMR as a One Health issue globally. Our study’s findings will add to the limited literature on this topic in the veterinary setting especially from Southeast Asia. Future research is needed to explore the most effective message types and the message delivery systems which address pet owners’ expectations for antibiotics in veterinary practices.

In summary, pet owners with poor knowledge of antibiotic use and with prior usage of antibiotics were more likely to expect antibiotics for their pets. Additionally, pet owners who expected antibiotics to be prescribed for their pets were more likely to receive it. This highlights the importance of effective communication by veterinarians to mitigate pet owners’ expectations to reduce inappropriate antibiotic prescribing.

## Data Availability

The raw data supporting the conclusions of this article will be made available by the authors, without undue reservation.
