# Supplementary material for: Expectation of antibiotics amongst owners of dogs and/or cats during non-routine visits to veterinary clinics in Singapore: a cross-sectional study
Source: Front Vet Sci. 2024 Nov 18;11:1491054. doi: 10.3389/fvets.2024.1491054 (PMC11609168; doi:10.3389/fvets.2024.1491054)
Supplement: Supplementary file 1 [file Table_1.DOCX]

**Questionnaire**

Thank you for participating in our survey. This questionnaire consists of 3 related sections, with each section focusing on different aspects of antibiotics and antibiotic resistance.

Q1. How long have you been a cat/dog owner? __________________ years __________________ months

Q2. How many pet cats/dogs have you ever owned? __________________

Q3. How many pet cats/dogs do you currently own?

Cats: __________________

Dogs: __________________

| **Section A**  *This section seeks your views on antibiotic use and antibiotic resistance for your pet cat or dog.*  *Please* ***TICK*** *the response category that applies to you.* |
| --- |

**Use of Antibiotics**

Q4. Has your pet cat/dog ever taken antibiotics (oral, intramuscular [injection], intravenous [drip], topical [cream/ointment/lotion/ear or eye drops] etc.)?

|  | Please tick ONE response |
| --- | --- |
| Yes | - 1 |
| No (Skip to Q9) | - 2 |
| Don’t know whether my pet has ever taken antibiotics, for e.g. the seller or groomer might have given my pet an antibiotic without my knowledge (Skip to Q9) | - 3 |

Q5. When did your pet cat/dog last take antibiotics (oral, intramuscular [injection], intravenous [drip], topical [cream/ointment/lotion/ear or eye drops] etc.)?

|  | Please tick ONE response |
| --- | --- |
| In the last month | - 1 |
| In the last 6 months | - 2 |
| In the last year | - 3 |
| More than a year ago | - 4 |
| Cannot remember when | - 5 |

Q6. What form(s) of antibiotics has your pet cat/dog ever taken?

|  | Please tick ALL that applies |
| --- | --- |
| Tablets/capsules | - 1 |
| Oral liquid | - 2 |
| Injection | - 3 |
| Intravenous (IV) drip | - 4 |
| Topical e.g. cream/ointment/lotion/ear drops/eye drops | - 5 |
| Others (Please specify): _______________________ | - 6 |
| Administration route not known / Unsure | - 7 |

Q7. Have you ever gotten advice from a veterinary practitioner (vet), veterinary technician or veterinary nurse on how to administer the antibiotic(s) for your pet cat/dog?

|  | Please tick ONE response |
| --- | --- |
| Yes, I have received advice on how to administer the antibiotic(s) (e.g. for how many times a day, for how many days) | - 1 |
| No | - 2 |
| Cannot remember | - 98 |

Q8. For what reason(s) did your pet cat/dog receive antibiotics?

|  | Please tick ALL that applies |
| --- | --- |
| To treat a bite wound or other skin wound | - 1 |
| To clear the build-up of plaque and tartar on my pet’s teeth or for a routine dental scaling | - 2 |
| To treat cancer or tumour(s) on the skin | - 3 |
| To treat a viral disease e.g. parvovirus infection, herpes virus infection | - 4 |
| To manage metabolic diseases e.g. diabetes mellitus, hyperthyroid, Cushing’s Disease | - 5 |
| To treat osteoarthritis | - 6 |
| To treat an urinary tract infection | - 7 |
| To treat an eye condition, e.g. cataract, glaucoma | - 8 |
| After a routine sterilization/desexing surgery | - 9 |
| To manage obesity issues | - 10 |
| Others (Please specify): _______________________ | - 11 |

Q9. On the scale shown, how much do you agree with the following statements on antibiotic use for your pet cat/dog?

|  | Strongly Disagree | Disagree | Neither Agree Nor Disagree | Agree | Strongly Agree |
| --- | --- | --- | --- | --- | --- |
| 1. I normally keep antibiotic stocks for my pet at home in case of emergency | - 1 | - 2 | - 3 | - 4 | - 5 |
| 1. If my pet is sick, I will usually give my antibiotics to it | - 1 | - 2 | - 3 | - 4 | - 5 |
| 1. I will save leftover antibiotics for future use for my pet | - 1 | - 2 | - 3 | - 4 | - 5 |
| 1. I normally stop giving my pet antibiotics when it starts feeling better or when symptom(s) subside | - 1 | - 2 | - 3 | - 4 | - 5 |
| 1. I will see another veterinary practitioner (vet) if my pet’s vet does not give it antibiotics | - 1 | - 2 | - 3 | - 4 | - 5 |
| 1. I will give my pet leftover antibiotics when I think it needs them | - 1 | - 2 | - 3 | - 4 | - 5 |

**Knowledge of Antibiotic Use for Pet Cats and Dogs**

Q10. When do you think you should stop giving antibiotics to your pet cat/dog once it has begun treatment?

|  | Please tick ONE response |
| --- | --- |
| When it feels better or when symptom(s) subside | - 1 |
| When it has taken all of the antibiotics as directed | - 2 |
| Don’t know | - 99 |

Q11. Please indicate whether you think the following statements are ‘True’ or ‘False’.

|  | True | False | Don’t Know |
| --- | --- | --- | --- |
| 1. Antibiotics can treat bacterial infections in pet cats/dogs | - 1 | - 2 | - 99 |
| 1. Antibiotics can treat viral infections in pet cats/dogs | - 1 | - 2 | - 99 |
| 1. Antibiotics should always be prescribed for respiratory tract infections for pet cats/dogs | - 1 | - 2 | - 99 |
| 1. Pet cats/dogs need antibiotics after a routine sterilization or desexing surgery | - 1 | - 2 | - 99 |
| 1. An abscess (i.e. a tissue cavity filled with pus) from a bite wound will usually heal without antibiotics | - 1 | - 2 | - 99 |
| 1. Antibiotics do not have side effects in pet cats/dogs | - 1 | - 2 | - 99 |
| 1. Bacteria can become resistant to antibiotics used in pet cats/dogs | - 1 | - 2 | - 99 |
| 1. The more antibiotics we use in society, the higher the risk that antibiotic resistance develops | - 1 | - 2 | - 99 |
| 1. The use of antibiotics in pet cats/dogs can reduce the effectiveness of antibiotics in humans | - 1 | - 2 | - 99 |
| 1. It is okay to use antibiotics for my pet cat/dog that were given to another pet cat/dog, as long as they were being used to treat the same illness. | - 1 | - 2 | - 99 |
| 1. It is okay to buy the same antibiotics or request them from a veterinary practitioner (vet), if they helped my pet cat/dog get better previously when it had the same symptoms | - 1 | - 2 | - 99 |

**Understanding of Antimicrobial Resistance in Pet Cats and Dogs**

Q12. Please indicate whether you think the following statements are ‘True’ or ‘False’.

|  | True | False | Don’t Know |
| --- | --- | --- | --- |
| 1. Antibiotic resistance occurs when a pet cat/dog’s body becomes resistant to antibiotics and they no longer work as well | - 1 | - 2 | - 99 |
| 1. Many infections in pet cats/dogs are becoming increasingly resistant to treatment by antibiotics | - 1 | - 2 | - 99 |
| 1. If bacteria are resistant to antibiotics, it can be very difficult or impossible to treat the infections they cause in pet cats/dogs | - 1 | - 2 | - 99 |
| 1. Antibiotic resistance in pet cats/dogs is an issue that could affect me or my family | - 1 | - 2 | - 99 |
| 1. Antibiotic resistance in pet cats/dogs is an issue in other countries but not here | - 1 | - 2 | - 99 |
| 1. Antibiotic resistance is only a problem for pet cats/dogs which take antibiotics regularly | - 1 | - 2 | - 99 |
| 1. Bacteria which are resistant to antibiotics can be spread from pet cats/dogs to their owners | - 1 | - 2 | - 99 |
| 1. Bacteria which are resistant to antibiotics can be spread from owners to their pet cats/dogs | - 1 | - 2 | - 99 |
| 1. Antibiotic-resistant infections could make medical procedures like surgery much more dangerous for pet cats/dogs | - 1 | - 2 | - 99 |

| **Section B**  *Please* ***TICK*** *the response category that applies to you.* |
| --- |

*For the following questions, please consider your pet’s* ***most recent consultation with the veterinary practitioner (vet)***.

Q13. What was the reason for your pet cat/dog’s last consultation with the veterinary practitioner (vet)?

|  | Please tick ALL that applies |
| --- | --- |
| For the treatment of bite wound or other skin wound | - 1 |
| For dental procedure e.g. clearing of plaque and tartar build-up, routine dental scaling | - 2 |
| For treatment of cancer or tumour(s) on the skin | - 3 |
| For the treatment of a viral disease e.g. parvovirus infection, herpes virus infection | - 4 |
| For the management of a metabolic disease e.g. diabetes mellitus, hyperthyroid, Cushing’s Disease | - 5 |
| For the treatment of osteoarthritis | - 6 |
| For the treatment of an urinary tract infection | - 7 |
| For the treatment of an eye condition, e.g. cataract, glaucoma | - 8 |
| For a routine sterilization/desexing surgery | - 9 |
| For the management of obesity issues | - 10 |
| Others (Please specify): _______________________ | - 11 |

Q14. At that last consultation with the veterinary practitioner (vet), did you request for antibiotics for your pet cat/dog?

|  | Please tick ONE response |
| --- | --- |
| Yes | - 1 |
| No | - 2 |
| Cannot remember | - 98 |

Q15. At that last consultation, were you expecting antibiotics for your pet cat/dog from the veterinary practitioner (vet)?

|  | Please tick ONE response |
| --- | --- |
| Yes | - 1 |
| No | - 2 |
| Cannot remember | - 98 |

Q16. Did your pet cat/dog receive antibiotics from the veterinary practitioner (vet) during that consultation?

|  | Please tick ONE response |
| --- | --- |
| Yes (Respond to Q17a) | - 1 |
| No (Respond to Q17b) | - 2 |
| Cannot remember (Respond to Q17b) | - 98 |

Q17a. How did you feel about the decision to administer antibiotics for your pet cat/dog at that time?

|  | Please tick ONE response |
| --- | --- |
| Extremely dissatisfied | - 1 |
| Dissatisfied | - 2 |
| Neutral | - 3 |
| Satisfied | - 4 |
| Very satisfied | - 5 |

Q17b. How did you feel about the decision not to administer antibiotics for your pet cat/dog at that time?

|  | Please tick ONE response |
| --- | --- |
| Extremely dissatisfied | - 1 |
| Dissatisfied | - 2 |
| Neutral | - 3 |
| Satisfied | - 4 |
| Very satisfied | - 5 |

| **Section C**  *We need to find out a little about your background to help us better understand the information you shared with us. All detail in this questionnaire will be kept strictly confidential.*  *Please* ***TICK*** *the response category that applies to you.* |
| --- |

Q18. What is your residency status?

|  | Please tick ONE response |
| --- | --- |
| Singapore Citizen | - 1 |
| Permanent Resident (PR) | - 2 |
| Foreigner | - 3 |

Q19. What is your birth year? __________________

Q20. What is your ethnicity?

|  | Please tick ONE response |
| --- | --- |
| Chinese | - 1 |
| Malay | - 2 |
| Indian | - 3 |
| Others | - 4 |

Q21. What is your gender?

|  | Please tick ONE response |
| --- | --- |
| Female | - 1 |
| Male | - 2 |

Q22. What is your current marital status?

|  | Please tick ONE response |
| --- | --- |
| Never married | - 1 |
| Married | - 2 |
| Separated | - 3 |
| Divorced | - 4 |
| Widowed | - 5 |

Q23. What is your current work status?

|  | Please tick ONE response |
| --- | --- |
| Full-time work | - 1 |
| Part-time work | - 2 |
| Looking for work | - 3 |
| Retired | - 4 |
| Homemaker | - 5 |
| Unemployed (able to work) | - 6 |
| Unemployed (unable to work due to medical conditions or disabilities) | - 7 |
| Student | - 8 |
| Others (Please specify): _______________________ | - 9 |

Q24. What is your highest educational qualification?

|  | Please tick ONE response |
| --- | --- |
| Below Secondary | - 1 |
| Secondary | - 2 |
| Post-Secondary (Non-Tertiary) | - 3 |
| Diploma and Professional Qualification | - 4 |
| University | - 5 |
| Post-Graduate Degree | - 6 |

Q25. Please select the fields in which you have ever undertaken any study/and or work.

|  | Please tick ALL that applies |
| --- | --- |
| Human health | - 1 |
| Animal health | - 2 |
| Agriculture or animal production | - 3 |
| Scientific research | - 4 |
| Scientific education | - 5 |
| NONE of the above | - 6 |

Q26. Which best describes your current residence?

|  | Please tick ONE response |
| --- | --- |
| HDB 1- & 2-room flats | - 1 |
| HDB 3-room flats | - 2 |
| HDB 4-room flats | - 3 |
| HDB 5-room & Executive flats/condominiums | - 4 |
| Condominiums & Other apartments | - 5 |
| Landed properties | - 6 |
| Others (Please specify): _______________________ | - 7 |

This is the end of the questionnaire. Thank you very much for your time and kind cooperation. We have a small token of appreciation for you, upon collection of your completed questionnaire.

**Note:** Only questions relevant to the study are shown
