# Supplementary material for: Expectation of antibiotics amongst owners of dogs and/or cats during non-routine visits to veterinary clinics in Singapore: a cross-sectional study
Source: Front Vet Sci. 2024 Nov 18;11:1491054. doi: 10.3389/fvets.2024.1491054 (PMC11609168; doi:10.3389/fvets.2024.1491054)
Supplement: Supplementary file 2 [file Table_2.DOCX]

**Supplementary materials**

**Table S2A. Variable selection for final multivariable logistic regression model assessing factors associated with expectation of antibiotics amongst pet owners**

| **Model Number** | **Variables in the model** | **AIC** | **BIC** | **-2LL** | ***P*-value*^+^*** |
| --- | --- | --- | --- | --- | --- |
| M1 | Age, Gender, Ethnic group, marital status, Educational level, Own Dogs, Ownership duration | 955.746 | 993.430 | 939.746 | NA |
| M2 | Gender, Ethnic group, marital status, Educational level, Own Dogs, Ownership duration (removed Age from M1) | 957.487 | 990.461 | 943.488 | 0.053 |
| M3 | Gender, Ethnic group, marital status, Educational level, Own Dogs, Ownership duration, **Prior antibiotic use** | 921.580 | 959.264 | 905.578 | **<0.001** |
| M4** | Gender, Ethnic group, marital status, Educational level, Own Dogs, Ownership duration, Prior antibiotic use, **Knowledge of antibiotics** | 913.952 | 956.347 | 895.952 | **0.0019** |
| M5 | Gender, Ethnic group, marital status, Educational level, Own Dogs, Ownership duration, Prior antibiotic use, Knowledge of antibiotics**, AMR knowledge** | 914.080 | 961.185 | 894.080 | 0.171 |

^#^Model 2 was derived by excluding age due to high variance inflation factor (VIF) from Model 1.

+ Log likelihood-ratio tests between models M2 and M1, M3 and M2, M4 and M3, and M5 and M4 respectively

**Final model

**Table S2B. Variable selection for final multivariable logistic regression model assessing factors associated with receipt of antibiotics amongst pet owners**

| **Model Number** | **Variables in the model** | **AIC** | **BIC** | **-2LL** | ***P*-value*^+^*** |
| --- | --- | --- | --- | --- | --- |
| M1 | Expected antibiotics, Age, Gender, Ethnic group, Educational level, Own Dogs, Ownership duration | 943.150 | 980.834 | 927.150 | NA |
| M2 | Expected antibiotics, Gender, Ethnic group, Educational level, Own Dogs, Ownership duration (removed Age from M1) | 941.733 | 974.707 | 927.734 | 0.445 |
| M3** | Expected antibiotics, Gender, Ethnic group, Educational level, Own Dogs, Ownership duration, **Prior antibiotic use** | 873.950 | 911.634 | 857.950 | **<0.001** |
| M4 | Expected antibiotics, Gender, Ethnic group, Educational level, Own Dogs, Ownership duration, Prior antibiotic use, **Knowledge of antibiotics** | 873.153 | 915.548 | 855.154 | 0.095 |
| M5 | Expected antibiotics, Gender, Ethnic group, Own Dogs, Ownership duration, Prior antibiotic use, **AMR knowledge** | 875.949 | 918.344 | 857.888 | 0.976 |

^#^Model 2 was derived by excluding age due to high variance inflation factor (VIF) from Model 1.

+ Log likelihood-ratio tests between models M2 and M1, M3 and M2, M4 and M3, and M5 and M3 respectively

**Final model
